# European Public Health News

**DOI:** 10.1093/eurpub/ckac050

**Published:** 2022-06-01

**Authors:** Dineke Zeegers Paget, Iveta Nagyova, Dineke Zeegers Paget, Floris Barnhoorn

**Affiliations:** 1 EUPHA Executive director; 1 EUPHA President; 2 EUPHA Executive Director; 1 Deputy Director EPH Conference

In this European public health news, the focus is on the next generation of public health professionals. Nagyova et al. highlight the successes of EUPHA, including attention to the younger generation and including the next generation in our governance structure. Barrios Aparicio et al. from the regional office for Europe of WHO embrace youth by highlighting WHO’s commitment to having the next generation involved in decision-making. Finally, Barnhoorn presents the efforts of greening this year’s EPH Conference, as requested by the younger generation.

It is clear that we need to make sure that the younger (future) generation of public health professionals is (critically) involved in what we do and in what we need to do to make our future healthier.

## Celebrating 30 years of EUPHA

NagyovaIveta 1
EUPHA President
Zeegers PagetDineke 2
EUPHA Executive Director

This year, EUPHA is celebrating its 30th birthday. Established in 1992, EUPHA started as a small network of public health professionals, where coordination between countries and providing the evidence were top priorities. Over the years, EUPHA has developed in a strong, reliable, science-based European partner with an excellent reputation. EUPHA is able to provide the right evidence at the right place at the right time, has become a strong advocate for public health at European level and also has developed action-oriented tools, like the European public health week.

So how did we grow to what we are now? Without being exhaustive, we would like to highlight six decisive developments that boosted EUPHA’s visibility.

### The growth of the *E**uropean J**ournal of P**ublic H**ealth*

The first development was the continuous transformation of the European Journal of Public Health. Under great leadership of our editors-in-chief (including Johan Mackenbach, Martin McKee and Peter Allebeck), the impact factor of the EJPH has continued to increase. The strict peer reviewing process has ensured the scientific quality of articles published. And the addition of key points for each article has increased the visibility of the evidence for policymakers. At the beginning of this year, the EJPH went fully open access in order to reach all researchers in Europe and beyond with sound scientific evidence. Finally, with the decision to publish online only, EUPHA showcases the need for going greener.

### Including the future generation of public health professionals

In 2011, EUPHA office welcomed three enthusiastic interns from Rome, who were committed to reach the younger segment of public health professionals and students. This was the start of EUPHAnxt, a unique network within EUPHA that organizes specific activities for the younger public health workforce. EUPHAnxt now has over 3600 subscribers, liaises with numerous student organizations in Europe and has an official seat in the EUPHA Executive Council. At the conference, several activities have been put in place for the future generation: the Young Researchers Forum (organized by our partner ASPHER), the Ferenc Bojan session where younger professionals can present their work in front of a scientific committee and the abstract mentoring programme (allowing first-time submitters to have their abstract checked before officially submitting). For students, lower fees were set for each European Public Health Conference. And EUPHAnxt established the fellowship programme, where younger professionals/students receive extra mentoring if they assist in writing daily updates.

### The increase of theme- specific networks

The EUPHA sections are a very strong tool for EUPHA’s visibility on a large number of topics at European level. EUPHA sections increased from 4 in 1994 to 26 in 2022. Several sections combine the EUPHA network of public health professionals with other networks, such as virologists (EUPHA Infectious diseases control section), architecture and city planning (EUPHA Urban public health section) or health lawyers (EUPHA Law and public health section). This interdisciplinary work method has increased our collaboration, not just for the EPH Conferences, but also for the organization of webinars, mid-year conferences and projects.

### From EUPHA Conference to European Public Health Conference

This year is the 15th European Public Health Conference. In 2008, the first European public health conference was organized to replace the EUPHA conference. This was done to include a wider audience and a wider range of co-organizers. Since 2008, the European Commission, WHO Regional office for Europe (WHO/Europe), ASPHER, EuroHealthNet, EHMA, ECDC and the European Observatory all take part in the organization of the plenary programme. This has made not only the plenary programme more attractive for a wider audience, but also allowed new topics to be included in the parallel programme. The European public health conference is the biggest public health event in Europe.

### Multiple sources of income

One of the biggest worries of non-governmental organizations is the financial sustainability. Years ago already, EUPHA has slowly developed multiple sources of income to finance the ambitious yearly work plans. At the beginning of EUPHA, finances mainly came from membership fees. Nowadays, EUPHA has four sources of regular income: membership fees, the EPH Conference, the European Journal of Public Health and European projects. These multiple sources stabilized EUPHA as an organization and allowed EUPHA to continue to expand its activities.

### A strong and motivated EUPHA office

It is clear that the EUPHA office has contributed to the success of EUPHA. The staff—although small in numbers—has shown enormous commitment to EUPHA and its goals and have gone beyond the normal job descriptions. Without this dedication, EUPHA would not be where it is today.

Finally, the success of 30 years of EUPHA would not be possible without the commitment of our members guiding us along the way and making sure we do not forget that EUPHA combines European public health with public health in European countries.

## Nothing about us without us: embedding YOUth in health decision-making in the European Region

Lourdes Barrios Aparicio, Hilaire Armstrong, Zhanibek Yerubayev, Torgyn Issa, Ivana Todorovic, Nargilya Gasanova, Alihan Berdiyev, Mikhail Sameke, Iana Kirilova, Daria Podchinenova, Hans Henri P. Kluge (WHO/Europe); Laura Plešnar (International Federation of Medical Students Associations); Vedran Raguž (International Pharmaceutical Students’ Federation); Andrej Martin Vujkovac (International Youth Health Organization); Dorothea Dalig, Jeffrey V. Lazarus, Roberto Pérez Gayo (Nobody Left Outside); and David Ritchie (European Cancer League Youth Ambassadors).

Today, the world has the largest generation of young people (the United Nations understands adolescents to include persons aged 10–19 years and youth as those between 15 and 24 years for statistical purposes without prejudice to other definitions by Member States. Together, adolescents and youth are referred to as young people, encompassing the ages of 10–24 years^1^) in its history. A total of 1.8 billion are aged 10–24.^2^ This generation is unique: there is an unprecedented level of communication and interconnectedness and their fluency in social media makes them more visible than ever before.

The COVID-19 pandemic has disrupted all aspects of life and its effects have been experienced in different ways by different populations.^3^ Importantly, today’s youth will face the long-term economic and social consequences of the pandemic.^4^^,^^5^ However, young people’s response to the pandemic and other key political issues, show their capacity and ability to lead and drive change. For example, in March 2022, through the high-level meeting on health and migration in the WHO European Region, the International Federation of Medical Students’ Associations (IFMSA) participated in the Ministerial session with health ministers and representatives of the 53 Member States, where they had the opportunity to present a statement and discuss strategic priorities for health and migration. Overall, young people are not only participating in high-level international events, but they are also working at the community level, facilitating action and building awareness.^6^

Young people are diverse, representing different backgrounds, concerns, expectations and needs. They are experts of their own reality and experiences. They are unafraid to challenge the status quo and raise their voice on topics of importance to them. Therefore, young people are a valuable resource, and should be a key stakeholder in building a more inclusive and sustainable future for all.

Participation is a guiding principle of the Universal Declaration of Human Rights. Young people should have equal opportunities to actively participate in health decision-making, and at all levels, for them to feel empowered to play a role in their own development, to build respect and mutual trust between stakeholders and to normalize the presence of youth in engagement opportunities.

Yet young people are often excluded from the discussion, or their involvement is limited to non-participation models, even in engagement opportunities that pertain to them and their future. This results in feelings of not being respected, trusted or heard.

Youth participation is often undermined by social, economic and institutional barriers. Specific challenges include:

- Negative attitudes of adults towards young people’s capabilities.
- Financial limitations. When engagement is voluntary and unpaid it creates participation bias, with those often participating from higher socioeconomic backgrounds and not representing the population of interest.
- Not all organizations have the time, knowledge, capabilities or capacity to effectively engage with young people.

Overall, the diversity inherent to young people means that different communication and engagement approaches are required, to ensure no one is left behind.

Authentic and meaningful youth engagement should be youth-centred and aim to include as many voices as possible.^2^^,^^7^^,^^8^ Meaningful engagement of young people about health and well-being issues that are important to them contributes to improvements in health policies, programmes and services, and in turn, impacts on health and social outcomes.^6^ Young people are both beneficiaries of healthcare services and can be leaders of health and well-being efforts. The inclusion of young people in health-related matters which—directly or indirectly—affect their lives, leads to better understanding, and improved acceptance, awareness and compliance with numerous healthcare recommendations. For instance, smoking prevention and cessation campaigns cannot be successful without active youth engagement from the early planning stages.

When governments are exploring using different policy levers to implement various health reforms, the inclusion of a diverse range of youth in health policy processes and decision-making is critical and should be mainstreamed. For example, decisions regarding how a country’s health system is financed have short- and long-term consequences on youth, with greater impacts compared to other populations due to future health insurance payments and taxation requirements.

Additionally, the young healthcare workforce needs to be part of these processes, as they are the target audience when designing health policies and programmes and are equally engaged in the policy making process, as youth representatives and as health professionals. It is equally essential to expand young health professionals’ capacity and capabilities in leadership, strategic planning, advocacy, partnership building and management, and to provide them with professional opportunities that allow them to contribute and grow. Young healthcare professionals can also act as youth champions and engage with youth in community and healthcare facilities, breaking stigma and discrimination and driving systemic change.

### Youth and WHO/Europe

Youth are an important stakeholder for the WHO/Europe and should be recognized and acknowledged for their knowledge and leadership of health issues. As a special initiative of the WHO Regional Director for Europe, WHO/Europe is actively working to increase meaningful engagement with youth, with a vision to amplify and embed their voices and perspectives into all areas of work and decisions that affect young people in the Region, including in implementation of the European Programme of Work, 2020–25.^9^

In September 2021, WHO/Europe hosted its first Young People and Young Professionals Forum (YPYP Forum), a pre-event of the 71st session of the Regional Committee for Europe. The YPYP Forum was facilitated by WHO/Europe but led in partnership with the IFMSA, the International Pharmaceutical Students’ Federation and the European Medical Students’ Association. The forum led to the development of the Roadmap for Engagement with Younger People in the WHO European Region and YPYP reflections on the Pan-European Commission for Health and Sustainable Development.^10^ One of the key recommendations from the Roadmap is for WHO/Europe to form a youth network. Work is currently underway to establish this mechanism and structure, with a vision to expand the youth network to include not only young health professionals, but to include diverse youth representatives from across the Region, e.g. young people living with a disability or chronic disease; young people who live in remote areas or caught up in social disruption; those being marginalized, stigmatized or criminalized because of their sexual orientation, gender identity or ethnicity; those experiencing homelessness, violence, abuse or exploitation; migrants and refugees, and those without access to health services or social protection.^2^^,^^6^^,^^8^^,^^9^

To continue efforts in creating spaces for youth engagement, WHO/Europe in partnership with the Albanian Ministry of Health and Social Protection and the Office of the Minister of State for Youth and Children, and the Municipality of Tirana are hosting the *Tirana 2022 Health and Well-being Forum for Youth*. The Forum aims to bring together youth from across the WHO European Region to network, engage in dialogue and share best practice examples of youth led interventions that address key health and well-being issues of importance to young people. The forum will take place over 3 days, from 25 to 27 October 2022.

Whilst the voices of young people are already being sought through the individual programme areas within WHO/Europe, WHO’s capacity to be more receptive and responsive to the views and needs of young people must be strengthened. The establishment of the Roadmap, the youth network and other activities to follow, redefines WHO/Europe’s relationship with young people in the Region, and will provide them with increased opportunities to engage with WHO/Europe and decision makers on health and well-being issues.

## 15th European Public Health Conference, 9–12 November 2022, Berlin, Germany

BarnhoornFloris^1^ 1
Deputy Director EPH Conference

Greening the EPH Conference Berlin 2022

The COVID-10 pandemic forced us to go virtual for two consecutive years, both the World Congress on Public Health (WCPH 2020) and the EPH conference 2021 were held as fully digital events. With the steady return to ‘normal’ life across Europe, we are increasingly confident that we can actually host an in-person conference in Berlin in November. In terms of organizing eco-friendly conferences, we have had a modest carbon footprint for 2 years but it looks like we are going back to square one with a much bigger carbon footprint this year. The question arises as to whether we are doing enough to reduce our carbon footprint when hosting the in-person Berlin 2022 conference?

Evaluation of the WCPH 2020 showed that 51% of participants prefer to attend in-person conferences, only 10% prefer fully digital conferences, and 39% prefer hybrid conferences. This confirms again that in-person conferences are generally seen as important for sharing scientific knowledge amongst researchers and useful for networking activities and debates. The in-person event enables colleagues in the same field to catch-up with each other and makes new connections. For the younger generation of researchers, it is often a start to build up their professional network. The downside of in-person conferences is the environmental impact. For example, a 3-day conference has a carbon footprint of 455 tons of CO_2_ eq., with delegates’ travel being the main contributor to its impact. Fortunately, there are measures we can take to minimize the impact of the Berlin 2022 conference on the environment.

EPH Conference is committed to reducing the environmental impact of the conference and finding ways to be more environmentally responsible. To mention the most important initiatives and measures: avoiding air travel; serving locally grown vegetarian/vegan meals; reducing material use.

### Avoiding air travel

We encourage our delegates not to travel to Berlin by plane, but to travel by train instead. Berlin is located in the heart of Europe and is a convenient hub for rail connections all across the continent. Delegates can easily reach Berlin from almost every major city in an environmentally friendly way with the lowest possible CO_2_ footprint. No matter what direction they are coming from, even from the UK, delegates can start their conference attendance in a sustainable way. The modern Berlin Hauptbahnhof connects cities from every direction and is located in the centre of Berlin. The Bahnhof Zoo is located in the western centre of the city. Each station offers quick public transit into the city centre and to the hub27 Berlin, our conference venue. Delegates can now also reach Berlin by night train from many destinations. If a flight is unavoidable for the trip to Berlin, we encourage delegates to join the atmosfair project and make a contribution to compensate the emissions of their flight.

### Foods and drinks

We offer a variety of healthy options throughout the EPH Conference:

- Fresh vegetables and fruit are the basis of all meals. Our catering service gives priority to fresh fruits and vegetables that are locally produced and in season, minimizing delivery distances and fuel consumption.
- We encourage the choice for vegetarian lunch and dinner options.
- We have chosen leaner meats and sea food, rather than beef meat.
- We do not offer alcohol at the conference and have limited the availability of drinks that are high in free sugars. Water from water dispensers is available throughout the conference.

In particular the catering company, we contracted follows the following ecological principles:

- Use of re-usable tableware only. If disposable tableware, CCG supplies compostable tableware and wooden cutlery.
- Water from water dispensers is available throughout the conference.
- Dishwashers are equipped with reverse osmosis units to reduce water consumption.
- Care is taken to ensure that all new kitchen equipment is designed to conserve water and energy.
- Pre-packed individual portions are restricted as far as possible in order to reduce waste.

### Reduced material use

We have adopted eco-friendly practices in communicating with delegates:

- Abstract submission, registration and invoicing are fully web-based. The full programme is available on the EPH Conference App.
- Printouts of conference materials are avoided. By switching to ePosters printing of poster materials by delegates is avoided.
- The printed Programme Book is only available on request and against payment of a fee. It is printed on FSC Mix paper. FSC Mix means at least 70% of the wood in the product is from FSC-certified material or recycled material; and 30% is controlled wood from responsible sources.

### EUPHA's Green Club

EUPHA has established a working group of environmental experts and environment-conscious colleagues to advice both EUPHA and the EPH Conference Foundation in their efforts to reduce the environmental impact of the conference. The Green Club is intended to brainstorm and advice the organizers of the annual EPH Conferences on how to decrease the impact on the environment.
